# Recent advances in live cell imaging of hepatoma cells

**DOI:** 10.1186/1471-2121-15-26

**Published:** 2014-07-08

**Authors:** Sandeep Salipalli, Prafull Kumar Singh, Jürgen Borlak

**Affiliations:** 1 Centre for Pharmacology and Toxicology, Hannover Medical School, Carl-Neuberg-Str. 1, 30625 Hannover, Germany

**Keywords:** Live cell imaging, Fluorescence, Bioluminescence, Green Fluorescent Protein, Proximity ligation assay, Lipid droplet

## Abstract

Live cell imaging enables the study of dynamic processes of living cells in real time by use of suitable reporter proteins and the staining of specific cellular structures and/or organelles. With the availability of advanced optical devices and improved cell culture protocols it has become a rapidly growing research methodology. The success of this technique relies mainly on the selection of suitable reporter proteins, construction of recombinant plasmids possessing cell type specific promoters as well as reliable methods of gene transfer. This review aims to provide an overview of the recent developments in the field of marker proteins (bioluminescence and fluorescent) and methodologies (fluorescent resonance energy transfer, fluorescent recovery after photobleaching and proximity ligation assay) employed as to achieve an improved imaging of biological processes in hepatoma cells. Moreover, different expression systems of marker proteins and the modes of gene transfer are discussed with emphasis on the study of lipid droplet formation in hepatocytes as an example.

## Background

Microscopy has contributed immensely to our understanding of cellular structure and morphology. However, traditional microscopic tools provide only limited information in terms of dynamical processes occurring in a living cell which are of great interest for biomedical researchers. Advances in optical methods, *in vitro* culture systems and molecular biology led to the advent of live cell imaging techniques. This non-invasive technique provides better insight into the biological role of target molecules by allowing researchers to investigate the dynamic processes occurring in living cells in real time. The technique has many potential applications in various fields of biomedical science including developmental biology, cell biology and tumor biology and provides opportunity to study the dynamic behaviour of living cells in context to gene expression, protein-protein interaction, co-localization, cell division, chromosomal dynamics and intracellular transport of bio-molecules. The success of live cell imaging relies on various factors including the specific imaging system, climate controlling devices for cultured cells under investigation, construction of recombinant plasmid DNA, transfer and expression of candidate genes and/or fluorescent proteins in mammalian cells. These factors greatly influence the fluorescent/bioluminescent signals obtained from the cultured cells. The gene transfer methods should not only be efficient in delivery and in ensuring stable expression but at the same time should exert minimum toxic effects to the cultured cells. Furthermore, the chosen fluorescent or bioluminescent markers should be minimally phototoxic to the cells at their highest expression levels. Amongst the bioluminescent markers, ATP dependent and independent luciferases from various sources have been extensively used in imaging experiments [1,2]. The use of bioluminescent markers is not only limited to *in vitro* assays or live cell imaging but is also applied to *in vivo* molecular imaging experiments. Various lines of luciferase expressing transgenic mice and cells have so far been developed and are frequently employed in biomedical research, and a major breakthrough in the field of fluorescent protein imaging was the discovery of Green Fluorescent Protein (GFP) by Osamu Shimomura who received the Nobel prize in Chemistry in 2008 together with Martin Chalfie und Roger Tsien [3,4]. After the advent of GFP the technique of live cell imaging has taken a leap in understanding the detailed and complex cellular dynamics. Apart from GFP and its variants, many other fluorescent proteins have been isolated from a variety of sources and are successfully used in imaging experiments of various cell types and their organelles. In this regard, live cell imaging has been employed to study functional genetics of liver specific diseases including steatosis, which results from accumulation of lipid droplets in hepatocytes [5].

Efficient gene delivery in mammalian cells is another aspect of our review with appropriate choices of cell type specific promoters and their use for targeted gene delivery to hepatoma lines such as HepG2 and Hep3B. Nonetheless, the concept of gene transfer through plasmids started in bacteria via both, physical and chemical methods. Similar approaches have been used in hepatoma cells and other higher eukaryotes and mammalian cells and include lipofection, DEAE-dextran, calcium-phosphate, viral vectors, peptides and electroporation [6]. Lipofection has been used to achieve transient as well as steady transfection in hepatoma cells resulting in an improved and stable expression of transgenes even after several passages [7]. To develop protocols for cell type specific reporter activity, we discuss the use of alternate promoters and vectors for stable expression in actively dividing cells.

### Bioluminescent markers

Bioluminescence is the phenomenon of the production of light by a chemical reaction within a living organism. It was first discovered in firefly (*Lampyridae* species) and since then has been used for various screening and staining activities with an advantage of observing the cells under a compound microscope. Firefly luciferase (FLuc) emits luminescence (up to 560 nm) without the requirement of any external light excitation and uses ATP for the conversion of its substrate luciferin to oxyluciferin in a luciferase enzyme catalyzed oxidation reaction. Initially, FLuc was used only in luminometery based reporter assays using cellular lysates. Later luciferase expressing cells and mouse lines were developed for non-invasive imaging of rodents. Injection of the luciferin substrate in mice produces luminescent signals that can be easily detected by *in vivo* imaging modalities. Apart from beetle, luciferase has been isolated from members of the coelenterazine species, i.e. *Gaussia, Renilla, Pleuromamm and Oplophorus*. The luciferase from *Renilla sp.* (RLuc) uses a different substrate “coelenterazine” and produces a higher and stable luminescent signal as compared to the FLuc [1]. RLuc has an added natural advantage of being an ATP independent enzyme, and thus requires less energy to produce luminescence. However, a major limitation of FLuc and RLuc is their short life span and therefore these luminescent proteins cannot be used for long duration imaging assays. This led to the development of a more photostable and robust luciferase in the form of an enhanced beetle luciferase (ELuc) which again uses luciferin as the substrate and is an ATP dependent enzyme [8]. A comparison between ELuc and FLuc showed phenomenal differences in the intensity and photostability of the two luciferases. However, the Km values (Michaelis constant) of the enzymes in regard to ATP consumption were found to be almost equal, and it was concluded that the ATP use is not the reason for a better performance of Eluc. Moreover, ELuc was tested in cell lines such as NIH/3T3 using various promoters, and it was shown that the luciferase primarily localizes in nucleus, cytosol and in peroxisomes. Certain vectors and promoters also play an important role for their suitable expression namely pCMV vector used in mice cells along with the astrocyte specific promoter mPer2 which outperformed FLuc by a factor of 10- and 16-fold in cell extracts and live cells, respectively [8].

### Green fluorescent protein

In contrast to bioluminescence where the enzyme catalyzed reaction initiates the excitation of luminescent molecule, fluorescence is triggered when photons from an external source excites the light absorbing pigments. Green Fluorescent protein (GFP) was the first fluorescent protein to be discovered in jelly fish (*Aequorea victoria)*. Wild type GFP (wtGFP) is a 26.9 kDa protein with a major and a minor excitation peak (395 and 475 nm, respectively) and a single emission peak at 509 nm. The chromophore of this FP is formed by three amino acid residues consisting of ser65-tyr66-gly67 and held by a single α-helix surrounded by 11 β-barrel sheets to prevent its quenching by water [8].

Several modifications have been made to wtGFP to enhance its fluorescence intensity as well as stability by minimizing photobleaching. In the year 1995, Heim et al. [9] introduced a single point mutation at the S65T residue that resulted in enhanced fluorescence at the same emission spectra but with a shift in excitation peak from 395 nm to 488 nm. The mutation also significantly reduced the time required for formation of the fluorophore from 2 hours to 0.45 hours.

Several additional mutations in the protein helped the molecule to fold optimally at 37°C while the protein expression was improved by codon optimization of the wtGFP according to the host organism [10]. Additional mutants were generated replacing the Serine65 with threonine, alanine, glycine, cysteine or leucine leading to a protein with a single absorbance peak at ~489 nm [9-12]. Expression of GFP at high concentrations in the cells posed the problem of dimerization which was resolved by side directed mutations at A206K, L221K or F223R residues [13]. The enhanced GFP (EGFP) was developed with the modification and mutations described above at various positions of wtGFP. Proteins tagged with enhanced GFP can be visualized in cells with low light intensities causing less photobleaching to enable imaging and quantification of intracellular proteins and its pathways effectively [14-16]. To tackle the problem of mixed chromophores in live cell imaging experiments, several mutants of wtGFP including Enhanced GFP (EGFP) and Emrald GFP were generated which retained its fluorescent properties but with non-overlapping spectral properties [17].

Neutral chromophore GFP has also been generated with a shift in the excitation and emission wavelengths to UV and green light, respectively [12,18]. The proteins were again mutated to create Sapphire which displays increased emission at 37°C [10]. The new mutant of Sapphire called Turbo Sapphire has better pH-stability with UV excitation and thus has a larger separation between the excitation and emission wavelengths. Table 1 summarizes the development of different variants of FPs and their physical properties.

**Table 1:** Brief summary of various fluorescent proteins developed and their technical details

**Fluorescent protein**	**Mutations**	**Ex. λ (nm)**	**Em. λ (nm)**	**Photostability**	**Brightness (% of EGFP)**	**Reference**
**Green Fluorescent protein**						
wtGFP		395/495	509		48	
EGFP	F64L, S65T	488	509	++++	100	[19]
Sapphire	S72A, Y145F, T203I	399	511	++		[11]
T-Sapphire	Q69M, C70V, V163A, S175G	399	511	++	78	[20]
Emerald	F64L, S65T, S72A, N149K, M153T, I167T	487	509	+++	116	[21]
Superfolder	S30R, Y39N, F64L, (S65T/G65T), F99S, N105T, Y145F, M153T, V163A, I171V, A206V	485	510	+++	160	[22]
**Blue Fluorescent protein**						
BFP	Y66H, Y145F	360	442	+		
EBFP	BFP + F64L, S65T	380	440	+	27	[16]
EBFP 1.2	EBFP + S30R, Y36N, T65S, S72A, N105T, 117IV, N198S, A206V	379	446	++	53	[23]
EBFP1.5	EBFP1.2 + F145H, H148N, M153A	381	449	++	68	[23]
EBFP2	EBFP1.2 + I128V, V150, D155V, V224R	383	448	+++	53	[23]
Azurite	EBFP + T65S, V150I, V224R	383	447	++	43	[24]
Sirius	F46L, T65Q, W66F, Q69L, Y145G, H148S, and T203V, F223S	355	424	+++	12	[25]
**Cyan fluorescent protein**						
ECFP	Y66W, F64L, S65T	433/445	475/503	++	39	[26]
Cerulean	S72A, Y145A, H148D	433	475	+++	79	[27]
SuperCFP		433	474	++	45	
**Yellow fluorescent protein**						
EYFP	T203Y	513	527	++	151	[28]
Citrine	S65G, V68L,S72A, T203Y, Q69M	516	529	++	174	[29]
Venus	S65G, V68L,S72A, T203Y, F46L, M153T, V163A, S175G	515	528	++	156	[30]
Topaz	S65G, S72A, T203Y	514	527	++	169	[21]

Several fluorescent proteins from different species have been discovered with various excitations and emission spectra ranging from ultraviolet to far-red or infrared [10]. These have been used in several cells and organelles, and some of them are discussed in this review.

### Blue fluorescent proteins

Development of different variants of GFP with different absorption and emission spectra provided the opportunity for simultaneous multi-color staining of the cells. Early variants included the blue fluorescent protein (BFP), developed by introducing a point mutation (Y66H) to shift the absorbance and emission spectra to 384 nm and 448 nm, respectively, and cyan fluorescent protein (CFP) which was discovered from mutants of *A.victoria*[17]. Since the spectra of BFP and EGFP are distinguishable, a combination of these two FPs was first used for multi-color imaging in cells and Fluorescent (Förster) Resonance Energy Transfer (FRET) analysis [9,31,32]. However, a major drawback of BFP was its low intensity and faster photobleaching [33]. Therefore, enhanced BFP (EBFP) was developed by using codon optimisation for human cells [34]. Notably, Azurite, an EBFP, was developed by the Dauherty’s group and was reported to be 40-fold more photostable and brighter than the conventional BFP. EBFP was generated by incorporation of two additional point mutations (F64L and S65T) in the BFP and was considered to be the brightest BFP for the time until further improvements were made [24]. Subsequently, EBFP 1.2 was developed based on mutations at S30R, Y39N, T65S, S72A, N105T, I171V, N198S and A206V in EBFP and was 4-fold brighter than EBFP [23]. Further modifications of EBFP 1.2 led to the development of EBFP 1.5 which was more photostable than its predecessor EBFP 1.2 but with no increase in fluorescence. Azurite, as previously mentioned, had increased photostability as compared to EBFP 1.2 and EBFP 1.5 due to the mutations at V150I and V224R, respectively. However, this caused a decrease in the fluorescence by 30% [24]. Screening of a library of mutants generated by random mutagenesis identified EBFP2 with enhanced fluorescence and photostability when compared to EBFP and Azurite. EBFP2 was reported to be 4-fold brighter and 550-fold more photostable than EBFP and 1.4-fold brighter and 2.9-fold more photostable than Azurite [23]. However, both EBFP2 and Azurite tend to dimerize when expressed in cells at higher concentrations. This was overcome by replacing the alanine residue at position 206 by valine [35]. BFP was further modified to develop a stable red fluorescent protein (RFP). Site directed and random mutagenesis of RFP generated mTagBFP with a shift in excitation spectrum and blue emission [36]. Although mTagBFP is the brightest among all the BFPs produced, it is 1.4-fold less photostable than EBFP2 [23]. Therefore, mTagBFP was mutated at I174A to give rise to mTagBFP2, i.e. a 1.5 fold more photostable BFP with a similar spectral profile and maturation half time as that of the parent protein [37].

CFP and Yellow Fluorescent Protein (YFP) were developed as a multi-color pair. CFP was also brighter and more photostable when compared to BFP [9] and was created by a point mutation (Y66W) with a spectra intermediate between BFP and EGFP. Further improvements were also made to increase the stability and intensity of CFP [18,32]. Mutation at T203 position of wild type GFP with an aromatic amino acid resulted in shifting of excitation and emission over 20 nm into yellow wavelength. This led to the development of Enhanced Yellow FP (EYFP). Although EYFP is a dimer and sensitive to chlorine, three variants of EYFP were developed namely citrine (monomer), Venus - a fast maturing FP at 37°C and the yellow fluorescent protein for energy transfer (YPet) - used in FRET along with cyan FPs [29,30,38].

### Red fluorescent proteins

Red Fluorescent Protein (RFP) was first isolated from an Anthozoan, *Diascoma,* with excitation and emission of 558 and 583 nm, respectively [39]. The major advantage of RFPs over GFPs is their longer excitation wavelength causing less damage to the cells and much less auto fluorescence in cells at the red spectrum. Also the maturation of FPs obtained from anthozoan is more efficient as compared to that of jelly fish FPs at 37°C. Native RFP has the disadvantage of forming tetramers which at times tends to aggregate in the cells and also cause false oligomerization of target proteins to hinder their native function [40]. DsRed, the common RFP available, has prolonged maturation time often taking days to turn red from a greenish complex; this feature has been used to observe aggregate formation in target cells [41]. Monomeric RFPs have also been developed from DsRed through a series of mutations, and the resultant proteins exhibited different maturation rate as well as fluorescent properties, i.e. mOrange, mKO, mStrawberry, mCherry, Tag RFP, mPlum, mKate and tdTomato [42]. Due to its fast maturation rate, high photostability and wide pH tolerance mCherry is a widely used long wavelength FP. However, it tends to dimerize that limits its experimental use. Later the protein was modified to prevent its dimerization in the cells. It is considered to be the brightest RFP with enhanced photostability, and it has only a 3 nm longer excitation and emission wavelengths compared to its predecessors [43]. Similarly, Tag RFP was developed from wtRFP isolated from sea anemone *Entacmaea quadricolo* by introducing mutations at five different sites (R162E, Q166D, S180N, F198V and F200Y) [44].

### Fluorescent proteins and cell organelles

As of today, live cell imaging has advanced mainly due to the advent of FPs tagged biomolecules with specific functions in the cell. FPs have successfully been cloned into bacteria to mammalian cell lines either alone as a marker or along with a gene of interest as a fusion protein [45,46].

Several organic dyes have been tagged to cell organelles to visualize the dynamics under a microscope, however, not all dyes are readily accepted by the cell organelles and in such conditions FP tagged proteins are used to target the organelle. Multi-color live cell imaging is an effort to observe multiple organelles, their spatial organisation, interactions between organelles and their constituent components. Therefore, the successful application of the technique demands FPs and dyes with non-overlapping emission spectra [47].

In recent developments organelle staining dyes such as Hoechst 33342, MitoRed, DiOC_6_, SYTO 9 and rhodamine B were tested along with several other dyes for imaging of various organelles in live cells and for the study of host pathogen interactions [48]. In this regard, lipid droplet staining has been done since a long time but only with organic dyes such as oil red, and none of the FPs mentioned earlier have been used for successful imaging of lipid droplets. The different fluorescent proteins widely used for staining of different organelles of a live cell are listed in Table 2.

**Table 2:** Summary of fluorescent proteins used for various cell organelle staining

**Organelle**	**Fluorescent proteins**					**Reference**
	**EGFP**	**CFP**	**Tag RFP**	**mCherry**	**DsRed**	
**Nucleus**	Yes	Yes	Yes	Yes	Yes	[10,31]
**Mitochondria**	Yes	Yes	Yes	Yes	Yes	[29,31]
**Actin**	No	Yes	Yes	Yes	Yes	[41]
**Histones**	Yes	Yes	Yes	Yes	Yes	[45,46]
**Peroxisomes**	Yes	Yes	Yes	Yes	Yes	[45,46]
**Tubulin**	Yes	Yes	Yes	Yes	No	[13]
**Golgi**	Yes	No	Yes	Yes	No	[15]
**Endosomes**	Yes	Yes	Yes	Yes	Yes	[45,46]

### Fluorogen activating proteins

Fluorogen activating proteins (FAP) are proteins/peptides such as bovine serum albumin and IgG that can bind and activate the fluorescence of target fluorogen molecules and may also alter their spectral properties. Conjugation of 9-(2-carboxy-2- cyanoviny1)julolidine (CCVJ) fluorogen to either BSA and/or IgG not only improved its solubility but also the fluorescence intensity by 6.3-fold when compared to the unconjugated molecule [49]. The use of larger FPs such as GFP in reporter assays hinders the function of the fusion proteins in the cells. Therefore, small tag systems such as tetracysteine motif (CCXXCC) were genetically inserted into the target proteins that can bind with high specificity to biarsenical dyes, Resorufin Arsenical Helix binder (ReAsH) and FIAsH (Fluorescein Arsenical Helix binder) in live cells and lead to an enhanced fluorescent signal [50].

Of note, FlAsH, a derivative of 4’, 5’-bis (acetoxymercuri) fluorescein, is a cell permeable non-toxic ligand that binds specifically to four cysteine residues (I, i + 1, i + 4 and i + 5) of the target proteins α helical structure to emit fluorescence [51]. Out of the fourteen different FlAsH ligands screened, FlAsH-EDT2 showed strong affinity for hexamer peptides and positive emission at 635 nm. FRET studies in HeLa cells with FIAsH-EDT2 genetically fused to the COOH-terminus of an enhanced cyan fluorescent protein (ECFP-FIAsH) evidenced a 3-fold increase in fluorescence at 635 nm when compared to the ECFP alone transfected cells [32,51,52]. However, emission of ECFP at 480 nm declined to about 30% in FIAsH-EDT2 transfected cells due to FRET interactions. ReAsH-EDT2, a resorufin based fluorescent complex with excitation and emission maxima of 593 nm and 608 nm, respectively, was successfully tested for FRET studies with GFP and YFP probe pairs in HeLa cells [53]. Similar protein-ligand complexes were synthesized and used namely CrAsH-EDT2, sFlAsH-EDT2, F2FlAsH-EDT2, CHoXAsH-EDT2, SpLAsH-EDT2-Alexa594, CaG FlAsH-EDT2 and AsCy3-EDT2. Several modifications have been carried out to improve the quantum yield, tetracysteine binding affinity and to reduce the cytotoxicity of these complexes. Specifically, Malachite green (MG) and Thiazole orange (TO) derived intercalating FAPs were developed which bind to RNA aptamer and DNA, respectively, and lead to enhanced (2,360- and 550-fold, respectively) and bleaching stable fluorescent signal from the fluorogens [54-56].

**Figure 1:**
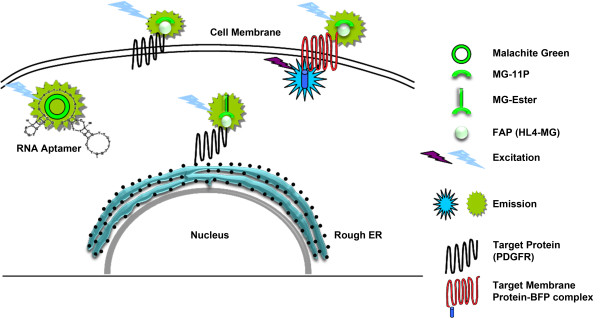
**Live cell imaging with fluorogen activating proteins (FAP).** Malachite green (MG), an organic dye and its membrane permeable (MG-Ester) as well as the membrane impermeable variants (MG-11P) are used in FAP imaging. Essentially, the FAP system consists of an encoded protein such as a single chain antibody fragments to activate the fluorescent dye and/or its derivatives. Note, initially MG was used to visualize RNA aptamers. The intracellular trafficking of the membrane bound receptor tyrosine kinase PDGFR is given as an example. Similarly, membrane bound proteins tagged with both FAP and fluorescent proteins like blue fluorescent proteins are useful in studying the protein positioning across the membrane [54].

Several unique membrane permeant and impermeant FAPs were developed by screening a library of human single-chain antibodies (scFvs) using derivatives of thiazole orange and malachite green. The screened clones have comparatively smaller size (the smallest being 110 amino acids long that is almost half the size of GFP) and thousands-fold higher brightness as compared to the typical FPs. Moreover, different spectral variants of MG and TO could be easily generated by different combination of the screened fluorogens and FAPs. The fluorescence and differential interference contrast imaging microscopy was performed in NIH3T3 cells with the membrane impermeable (MG-11p) and membrane permeable (MG-ester) forms of MG fluorogens and FAP HL4-MG scFv tagged to membrane protein platelet-derived growth factor receptor (PDGFR). The MG-ester signal was detected from the plasma membrane while MG-11p emitting fluorescence inside the cells suggested a localization near endoplasmic reticulum [54]. Figure 1 illustrates the uses of membrane permeable and impermeable MG FAP fluorogens and their application with a fluorescent tagged protein to study trafficking of a transmembrane protein.

Additionally, these FAPs were also tagged along with GFP and other fluorescent proteins to study plasma membrane and transporter proteins such as the insulin regulated glucose transporter (GLUT4), β2 adrenergic receptor (β2AR) and cystic fibrosis transmembrane conductance regulator (CFTR). These genes were designed such that the FAP and GFP were present as intracellular and extracellular reporters and the fluorogens, being membrane permeable and impermeable, were used to observe the mobility and placement of the target proteins tested in NIH3T3, C2C12 and HEK293 cells [57]. FAPs have been tested and proved to be reliable fluorescent markers for detecting and imaging various activities in a cell like protein motility studies and even for transmembrane protein activity. FAPs provide wide scope in developing alternatives to traditional fluorescent proteins where size hinders the activity of the target proteins.

### Vectors for transient and stable transfection in cell lines

Bacterial transformations have led to the expression of various genes in the prokaryotic system with certain limitations as compared to the eukaryotic cells. However, the transfer of genes in the mammalian cells requires much more sensitive approaches, and several methods have been developed for effective transfer and stable expression of genes in these cells. With the advent of viral vectors much progress has been made in the field of gene transfer.

### Promoters for hepatic cell lines

Expression of a foreign gene inside a cell greatly relies on the choice of the vector, mode of transfection (stable vs. transient) and type of promoter used (constitutive vs. cell-specific and inducible vs. non-inducible). The choice is made based on the experimental requirements, cell type and the technical expertise. To achieve stable transfection, integration of transfected genes in the host genome is desirable. The transient expression vectors reside in the cytosol without replicating and therefore the genes of interest are expressed for a limited period of time. In contrast, stable transfection vectors facilitate the integration of the transgene in the host cell genome and therefore, it is replicated and subsequently inherited. The introduction of a selection marker (antibiotic resistance gene) in the expression cassette allows the differentiation and expansion of the stable transfected population from the non-transfected cells.

The use of cell type specific promoters allows greater control over the expression of foreign genes. Liver cell specific promoters such as Glycerol-3-Phosphate Acyltransferase (GPAT I and GPAT II) and phosphoglycerate kinase 1 (pgk-1), albumin, human alpha1-antitrypsin and hemopexin are shown to be active in hepatocytes of human and mice origin [7,58,59]. Similarly, viral promoters and vectors have been tested in different cell lines for their specificity and stability of expression. pCNS and pCNS-D2 vectors were reported to express cloned genes in cell lines and can also act as a shuttle vector between bacteria and mammalian cell lines to facilitate an easy cloning process. This vector system has been tested for stable expression of luciferase in HepG2, Hep3B, HeLa, SNU638 and SNU668 cell lines. The luciferase expression was higher in HepG2 and SNU638 cells as compared to other cell lines.

Cytomegalovirus (CMV) and Simian virus 40 are known to infect human and other primate cells and have been shown to induce a stable expression of transgene in various cell lines [60]. During the study of CMV promoter systems, a 5’ Untranslated Region (5’ UTR) was identified which was shown to boost the expression of viral proteins facilitating the rapid propagation of viral particles in the host cell. Incorporation of UTR regions in the promoter of viral vectors boosted the expression of genes of interest in host cells such as CHO or HepG2. Notably, the use of two copies of the UTR regions further improved the expression of target genes in actively dividing cells [61].

**Figure 2:**
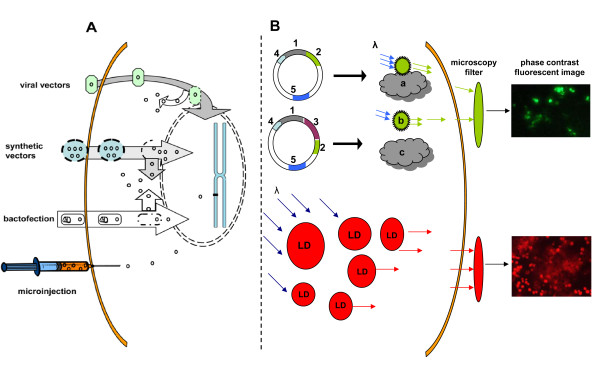
**Schematic overview of gene transfer methods for live cell imaging studies.** Depicted are methods for gene delivery and expression of fluorophore tagged proteins, **(A)** Transient and stable expressions of genes using viral or synthetic vectors, microinjection and lipofection. The later is the most frequently used method for gene transfer. Bacterial vectors of *Listeria* and *Salmonella sp* although hurdled by low transfection efficiency have been used for special cell lines. **(B)** Recombinant plasmid expressing fusion protein (target protein and fluorophore) under a common promoter (P) in host cells. Incorporation of a selection marker (SM) in the plasmid provides opportunity for controlled expression of the cloned genes. Upon excitation with specific wavelength, the fluorescence images are captured using appropriate filters. This provides the information on localization and probable interactions of the target protein with different cell organelles and/or other biomolecules. Cell organelles or structures can be stained using synthetic dyes such as Oil Red O for intracellular staining of lipid droplets. P: promoter, TP: target protein encoding gene and protein, FP: fluorescent protein encoding gene and protein, LD: lipid droplet stained with Oil Red O.

Later, the UTR regions were found to be the Internal Ribosome Entry Sequence (IRES) being responsible for uninterrupted expression of genes. IRES were first discovered in RNA of Poliovirus in 1988 by Pelletier and Sonenberg [61] and facilitate the viral replication machinery by providing means for viral RNA to bind with the 40s subunit of the host ribosomal complex thus eliminating the need for eukaryotic translation factors [62,63]. Since then, several viral vectors have been developed including the recently explored corona virus of the SARS virus family. This RNA based vector provides a stable and prolonged expression for more than 30 passages in different cell types [62]. Insertion of IRES site in expression vectors also offers the possibility of separate expression of the cloned genes under a single promoter (Figure 2) [61]. Table 3 summarizes the list of various promoters and plasmids that have been used extensively in mammalian cell lines.

**Table 3:** Promoters and vectors used for stable/transient expression in mammalian cells

**Expression in cell line**							
**Vector**	**Promoter**	**HepG2**	**Hep3B**	**CHO**	**HeLa**	**3 T3**	**Reference**
**pCNS/pCNS-D2**	**hCMV/T7**	nil	stable/transient	nil	stable	nil	[60]
**pGL3**	**GPAT**	stable	stable	nil	nil	nil	
**pLR/IRES**	**RSV**	nil	nil	nil	nil	nil	[62,63]
**pSV2βCat**	**SV2**	stable/transient	nil	stable/transient	stable/transient	stable/transient	[60]
**PCMVβcat**	**CMV**	stable/transient	nil	stable/transient	stable/transient	stable/transient	[60]
**Puast**	**SV 40**	**expressed genes in yeast**					[60]

Among all promoters used CMV and SV 40 are most efficient in a wide range of cell lines. Apart from these cell specific promoters such as pgk1 and GPAT enhance expression levels in hepatoma cell lines.

### Modes of gene transfer in cells

A variety of methods have been developed for a robust and efficient gene transfer without causing toxicity to the host cell.

Earlier physical methods such as electroporation and microinjections [64,65] were primarily used for gene transfer studies but have been sidelined gradually due to the obvious disadvantages of being time consuming and the associated technical difficulties. More advanced methods have come into place for gene transfer according to the need for expression as a plasmid entity or as an integrated system in genome which also can be used for gene knockouts.

### Viral vectors

Non-pathogenic viral vectors are commonly used for gene transfer studies. Baculovirus of the *Autographa californica multicapsid nucleopolyhedrovirus* (AcMNPV) and *Bombyx mori nucleopolyhedrovirus* (BmNPV) genus provide a safe and efficient means of gene delivery to target cells [66]. Baculoviral vectors have been modified to infect mammalian cell lines either as the whole baculovirus or as a helper cell mediated gene delivery in cells. Adenovirus terminal repeats were used for the replication of the target gene, and the whole plasmid was packed into the baculoviral vector Bac-B4 for transfer in cell lines such as 293B5B1. The transfected cells showed not only stable expression but also a 100-fold increase in the vector titer during subsequent passages [67]. Of note, DNA fragments of approximately 38 kb were efficiently transferred into different cell lines using such a vector. Direct incubation of oligodendrite and chicken muscle cells with baculoviral vector showed a transfection efficiency of 60% [68]. Further increase in transfection and expression efficiency was achieved by additional modifications in baculoviral vectors such as an introduction of the metallothione promoter (Bac-ME). The Bac-ME vector showed a transfection efficiency of 90% with low leaky expression and reduced cytotoxicity in HeLa cells. The expression efficiency of these vectors was further enhanced by treating the transfected cells with ZnSO_4_[69].

Although the baculoviral vectors were successfully used for gene transfer experiments in several mammalian cell lines they were replaced by other viral vectors and methods due to the associated cytotoxicity specifically at higher cell to virus ratio. Hence, non-viral vectors were developed for transfection of viral sensitive mammalian cell lines [70].

### Bacterial based methods

Bacterial based transfection has also been employed by cloning sequences belonging to the pathogenic bacteria E.coli. The modified genes from pathogenic bacteria like *Listeria monocytogenes* have also been used where the bacteria with its intracytoplasmic capabilities propagate into the host. Likewise, E.coli was modified so that self-lysis genes become activated as soon as the bacteria enter the host cell to release the vector into host cytoplasm [70].

Other bacterial methods involve T4SS, a *Bartonella sp.,* for the introduction of single strand DNA into the host cell. Thus, after entry the bacteria releases ssDNA into cytosol and along with the enzyme relaxase or integrase, which is bound to target DNA, starts to replicate to finally produce plasmid DNA as a whole by joining the loose ends present [70]. This mode of transfer is considered to be hazard free and is employed for a variety of cell lines.

Further methods of nonviral based gene transfer have been developed for higher efficiency and less toxicity to the cells. As mentioned earlier for efficient transfection the gene has to be integrated into the genome (constitutively) or is continuously expressed in the presence of a specific inducer reagent for the chosen promoter (inducible). Owing to variable results the need remains to develop efficient methods as has been attempted in the form of a dual plasmid approach using the mice myogenic C2C12 cell line. According to this method T-Rex plasmid system is first transfected into the cell line using a lipid based method with an efficiency of 70% and is activated by tetracycline. Thereafter, a modified T-Rex plasmid became available. After 18 days of initial transfection a second plasmid can be introduced with 40% efficiency that permits the study of complex biochemical reactions involving different factors [71].

In Table 4 the various transfection efficiencies of different methods are summarized. Bactofection seems to be an effective mode of transfection in cells without the risk of cytotoxicity as compared to some viral methods but much improvement has to be made for application of bactofection for a wide variety of cell lines.

**Table 4:** Summary of transfection efficiency in different cell lines through various methods

**Transfection efficiency**						
**Transfection method**	**HepG2**	**Hep3B**	**CHO**	**HeLa**	**3 T3**	**Reference**
**Baculovirus**	60-90%	60-90%	60-90%	60-90%	60-90%	[58,68-70]
**Baculovirus mediated adenoviral vector**	-	-	40-50%	55-60%	-	[67]
**Lipofection**	40-60%	40-60%	60-70%	60-70%	50-60%	[72]
**Cationic lipids**	47-60%	45-55%	65%	60%	-	[73,74]
**Non-viral**	-	-	-	60%	-	[71,75]
**E.coli (modified *Salmonells/Listeris sp*.)**	-	-	-	50%	-	[70]
**T4SS ( *Bartonella sp. *)**	-	-	-	50%	-	[70]
**T4SS ( *Bartonella sp. *)**	60-70%	50%	>70%	>60%	50-60%	[76-78]

### Lipofection

Lipofection involves formation of a positively charged lipid-nucleic acid complex by suspending negatively charged DNA/RNA molecules with cationic lipids for a short duration of time. The positive charge on the complex facilitates attachment to the cell membrane and entry via endocytosis [70]. A comparative study concluded that lipofection is the safest method to transfer genes into in mammalian cells amongst the various transfer methods [70]. Different commercially available kits with a broad range of efficiency on different cell lines are available. The transfection efficiency of methods varies depending upon the cell type, cell number and ratio of DNA and reagent used. Using lipofection and as determined with a GFP marker an efficiency of about 60% was obtained in CHO-K1 cell lines [72].

### Lysomotropic agents

The major challenge faced by most of the non-viral delivery systems is the degradation of the transfected nucleic acid in the endosomes and/or lysosomes as they are mainly internalized by endocytosis. Lysosomotropic agents like chloroquine and polyvinylpyrolidone (PVP) have been used effectively to increase the transfection efficiency of different delivery methods by reducing the lysosomic degradation of the transfected nucleic acid [73,79-83]. However, the cytotoxicity of these agents raises concerns against their use. Chloroquine is known to increase the intracellular pH leading to cytotoxicity and degradation of transfected DNA at higher concentrations and at prolonged exposure times (>4 hours). Amongst the two variants of PVP, PVP10 was reported to be cytotoxic at all concentrations while PVP40 showed negligible cell toxicity and even improved the transfection efficiency. PVP40 has been used for transfection studies in macrophages and hepatocytes [84]. Sucrose is also known to cause intracellular swelling of vesicles in endosome and lysosomes due to osmotic pressure [85,86]. Sucrose in conjunction with lipid-DNA complex or lipofection reagents was shown to increase their transfection efficiency in fibroblast cell. Furthermore, no toxicity has been reported using sucrose as lysosomotropic agent and a concentration range of 5 to 500 mM was safe in various cell lines such as CHO, COS7 and HEK 293 T cells.

Beneficial effects of these lysosomotropic agents were compared against Lipofectamine 2000 in COS7, CHO and HEK 293 cell lines. Essentially, the transfection efficiency of lipofectamine was increased by 6- and 3-fold when used in conjunction with PV40 and sucrose, respectively. Chloroquine, although being toxic showed an increase of 3- to 6-fold when used at a concentration range of 0.01 to 0.1 mM as compared to lipofectamine alone [87].

Poly(ethyleneimine) is another cationic polymer transfection reagent frequently used in a variety of cell lines. However high cytotoxicity is the major drawback associated with PEI and therefore several changes in its molecular structure have been done to reduce its cytotoxicity and this includes PEGylation and the introduction of carbohydrate, lipid and peptide moieties [75,88-90]. A recent study showed the improved transfection efficiency and low cytotoxicity of an anionic glycopolymer derivative of PEI in HepG2 and HEK 293 T cells [91].

### Modified lipids

Modified lipids were developed to enhance the transfection efficiency of vectors. Specifically, cationic oligopeptide lipids are charged lipids with a linker molecule to provide cations and are said to be more efficient in the presence of the serum containing medium [74]. Different ratios of nucleic acid and lipids were tested ranging from 1:1 to 9:1 and the ratio of 3:1 was found to be optimum in terms of cell viability and transfection efficiency [74]. Other synthetic vectors have been tested for gene transfer in various cell lines. Polyspermine Imidazole-4, 5-amide complex (PSIA) was employed with HepG2 as well as Cos 7 cell lines for gene targeted therapy. PSIA transfection efficiency is of only 20-30% in HepG2. Apart from the risk of PSIA degradation that hinders complex formation with plasmid DNA the process of PSIA synthesis requires strict environmental (pH and temperature) conditions before DNA can be introduced [92].

### Hepatocyte/hepatoma cells specific transfection

To improve transfection efficiency in various hepatoma cell lines like Huh-7, HepG2 and Hep3B different strategies were employed including lipofection, electroporation and microinjection. Microinjection (100 μg/ml) and lipofection (1 μg) methods were used for transfection of plasmid encoding fluorescent protein (pEGFP and pEYFP) tagged to human and mouse ADRP genes in Huh-7 cells as to study the pathways of lipid droplet metabolism. A transfection efficiency of 66% was achieved, and none of the methods affected the expression or the interaction of proteins in cells proving their reliability and safety. However, the transfection efficiency of these methods varied inversely with the size of the plasmid [93]. In this regard, adenoviral vectors have been employed in HepG2 cells to study the function of genes such as the cytochrome P450 monooxygenases in drug induced hepatotoxicity. Notably, CYP1A2, CYP2D6, CYP2C9, CYP2C19 and CYP3A4 were transfected in HepG2 cells using recombinant adenoviral vector with a transfection efficiency of approximately 70% [94,95].

In most cases transfection or transduction of primary cells proof to be difficult for several reasons like the stable state of non-dividing or quiescent cells. However, some viral vector-based methods have been used successfully such as the murine retroviral vector that can transfect primary hepatocytes stimulated by mitogens (epidermal or hepatocyte or keratinocyte growth factors). Moreover, the associated high costs and varying efficiency of the growth factors make these transfection methods unfavorable [96-99]. Lentiviral vectors are capable of transfecting quiescent cells [100-102] and have been successfully used for gene transfer experiments in various hepatoma cell lines [76,103,104].Although the discussed methods and markers are compatible with various if not all cell lines, the cell lines chosen have to be noted to select the right kind of vector and promoter system. An overview of the various transfection methods in different cell lines is given in Figure 2.

### Lipid droplet staining

**Figure 3:**
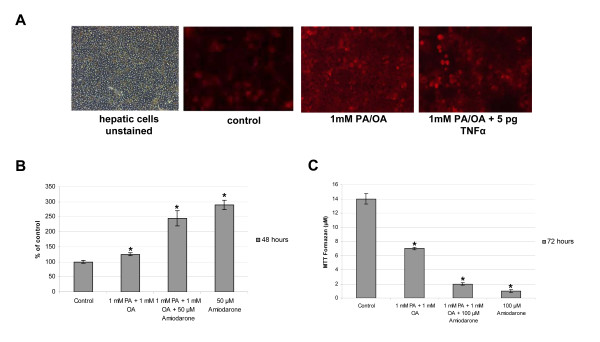
**Oil red O staining for imaging and quantification of lipid droplets in hepatocytes. (A)** Oil red O staining of lipid droplets in hepatic cells treated with fatty acid, amiodarone and/or TNF-α. **(B)** Spectrophotometric quantification of Oil red O stain to determine intracellular lipid load in hepatocytes treated with fatty acids and/or amiodarone. **(C)** MTT assay to determine cytotoxic effect of fatty acids, and/or amiodarone on cultured hepatocytes.

Research in recent years significantly advanced an understanding of the molecular causes of fatty liver disease, and live cell imaging of hepatoma cells proved to be extremely valuable for studying lipid droplet formation. Lipids are primarily stored in adipocytes as lipid droplets; however, under stressed conditions hepatocytes can also produce lipid droplets leading to pathological conditions like fatty liver disease [105,106]. Lipid droplet staining requires cell permeable dyes/fluorochromes which can bind specifically to the components of the lipid monolayer of droplets. The most commonly used dyes for lipid staining are Oil Red O, Nile Red and BODIPY with an excitation-emission spectrum in the range of 400–500 nm and 500–600 nm, respectively. Figure 3 depicts Oil red O staining of lipid droplets in cultured human hepatocytes treated with palmitic acid (PA) and oleic acid (OA) and the cardiovascular drug amiodarone which is known to cause steatosis and steatohepatitis in patients.

FPs and dyes with non-overlapping fluorescence spectrum are required for differential staining of multiple biological molecules involved in lipid droplet metabolism. To address this issue, a long wavelength FP and a short range dye Monodansyl Pentane (MDH) was developed for lipid droplet staining with excitation and emission in the range of 405 and 480 nm [106]. Such an approach allowed the study of perlipin 2 interactions on the lipid droplet as defined by the MDH stain. Notably, in the early works of Niemann et al. (2001) the use of MDH as a mean to study lipid droplets was reported [107]. MDH was also found to be photostable when compared to Nile red and BODIPY with a light emission of more than 15 minutes wherein the latter dyes were stable up to 10 minutes only [106]. MDH has a potential use for lipid droplet staining to facilitate an understanding of the formation of lipid droplets and their interaction with various proteins within hepatocytes.

The lipid droplet monolayer has many associated proteins namely Adipocyte Differentiation-Related Proteins (ADRP also known as perilipin 2/plin2) and Perilipins (Plins) which interact with other organelles in the cell. Simultaneous detection of lipid droplets and ADRP was achieved by transfecting HuH-7 cells with ADRP-GFP encoding plasmid and staining of lipid droplets with Oil red O. Although this method was useful in observing the cytoplasmic interaction of ADRP/plin2 with lipid droplets the precise localization of GFP-ADRP/plin2 at the surface of lipid droplet was distinctly observed only after incubating the cells with BODIPY558/568 dodecanoic acid, a fatty acid sequestered in lipid droplets as evidenced by imaging by confocal microscopy in live and fixed cells conditions [93]. While this study was one of the first reports on lipid-protein interactions investigated by live cell imaging the use of BODIPY hindered clear imaging due to background signals. To overcome these obstacles FRET and FRAP methods were used as a means to study these complexes.

Moreover, MDH stain provides an efficient stain for lipid droplets which allows proteins to interact with the droplets to be tagged with longer wavelength fluorescent proteins and also MDH provides a more stable and robust stain for lipid droplets as compared to the traditionally used Oil red O and Nile red stains.

### Lipid droplet-protein interaction in FRET and FRAP studies

#### ***FRET studies***

FRET is an approach to study protein-protein interaction and co-localization of organelles on the principle of energy transfer between two chromophores situated in close proximity (6–10 nm). The pair of fluorescent probes used has an overlapping spectral profile, and the emission wavelength of the donor probe (GFP) provides the required energy for the excitation of the acceptor probe (CFP). Acceptor bleaching can be used to demonstrate whether the organelles are within the FRET-distance (i.e. 6–10 nm), thus demonstrating true association on a molecular scale. FRET was used to observe motility of certain proteins as well as organelles [108].

Notably, laser scanning confocal microscopy based FRET assays have been employed to study the co-localization of lipid droplets and mitochondria in porcine oocytes using the Mitotracker Green (MTG) and Nile Red (NR) fluorochromes. FRET acceptor bleaching methods have also been used to examine mitochondria-lipid droplet co-localization [109].

**Figure 4:**
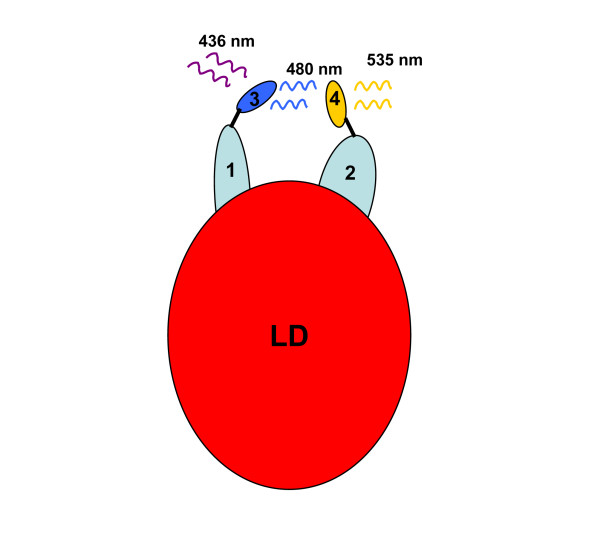
**FRET studies to determine interaction between plin5 and the ATGL protein on lipid droplet in adipocytes**[39]**.** Plin5 (1) and ATGL (2) proteins were tagged with FRET probes CFP (3) and YFP (4), respectively. The emission spectra of CFP act as excitation wavelength for the YFP. Detection of yellow fluorescence after activation of CFP (436 nm) suggest possible interaction of the two tagged proteins.

To study protein-lipid interactions on the surface of the lipid droplets, perilipin 2 was tagged with CFP and followed by cyan fluorescence. Since it forms a strong FRET interaction with the lipid stain 22-(N-(7-nitrobenz-2-oxa-1, 3-diazo) aminostearic acid (NBD), the co- localized protein-lipid gave a signal of yellow-to-orange. The mouse perilipin 2 gene was cloned into the mammalian expression vector pECFPN1 and was stably expressed in mouse L fibroblast cells. Subsequently, NBD was used to stain phosphatidyl choline to form NBD-PC complex, and a confocal microscopy based FRET assay was employed to determine the interaction between lipids and proteins in the cell. Similarly, co-localization of perilipin 2 and sphingomyelin (SM) near plasma membrane was demonstrated using NBD-SM and plin2-CFP vector constructs [110]. Although CFP and YFP are most widely used probes YFP has the disadvantage of having low quantum yield and poor signal to noise ratio hampering the process of individual CFP intensity measurement. To counter this problem, two ECFP molecules were fused; however, the result was a further decrease in the intensity due to multiple excited states causing interconversion of excited states by homotransfer. Notably, multiple excited states are due to two different crystal structure conformations of ECFP [111]. This problem was resolved by site directed mutagenesis of the His148 residue to aspartate, and the fluorescence intensity of the mutant was further increased by a factor of 2.5 as compared to ECFP. Further mutations at S72A and Y145A residues boosted the extinction coefficient of the monomer but caused a decrease in the quantum yield. The modified ECFP is known as Cerulean and is proved to be a better FRET donor than the conventional ECFP [111]. Figure 4 depicts a basic understanding on how FRET analysis can be done using ECFP and EYFP particularly for lipid droplet studies.

#### ***FRAP studies***

Fluorescent Recovery After Photobleaching (FRAP) is used to study single membrane bound structures, single cells and protein-lipid interactions. FRAP uses the concept of activating a fluorescent protein observing its intensity and subsequent bleaching by excess light to finally measure the bleached region for binding activity to the surface of the secondary structure or membrane. CFP and YFP have been commonly used to study the interactions of perilipin 1 (plin1) and perilipin 5 (plin5) with Adipocyte Triglyceride Lipase (ATGL) and its corresponding activator α-β-hydrolase domain-containing 5 (Abhd5). On the basis of FRAP studies in COS 7 cells, it was concluded that plin5 interacts with ATGL but not plin1 [27].

ADRP/plin2 is shown to be located at the surface of cytoplasmic lipid droplets [112], and localization studies for DNA fragments encoding human and mouse ADRP were ligated in-frame to the 3’ end of EGFP, thereby generating GFP-hADRP (expressed by plasmid pLA4) and GFP-mADRP fusion products. To examine whether the GFP-ADRP products for both species retained the capacity to associate with these structures, constructs expressing the fusion proteins were transfected in HuH-7 cells. After stimulation with fatty acid, the formation of lipid droplets in HuH-7 cells can be identified by staining with lipophilic dyes and by antisera raised against ADRP/plin2. The signal from GFP-mADRP was detected from the surface of spherical intracellular structures while EGFP was seen throughout the HuH-7 cells. The localization of endogenous hADRP and the GFP-mADRP products coincided, indicating that GFP-mADRP was located at the surface of lipid droplets therefore demonstrating that it is possible to detect tagged and untagged ADRP on the same lipid droplet. To verify the localization of GFP-mADRP, cells expressing the fusion protein were incubated with BODIPY 558/568 dodecanoic acid, a fluorescent fatty acid analogue that is sequestered in lipid droplets. The dye was selected because of its spectral properties which allow differentiation from EGFP and a better cell permeability as compared to Oil red O. Results in both live cells and paraformaldehyde fixed cells showed localization of GFP-mADRP at the surface of lipid droplets stained with the BODIPY dye. Attempts to fuse EGFP to the C terminus of ADRP/plin2 yielded few cells that produced fluorescence of the chimeric protein but in this case the signal did not derive from the surface of lipid droplet. Consequently, EGFP fused to the N terminus of human and mouse forms of ADRP/plin2 was used in further studies [93].

It is well recognized that caveolins are found on lipid droplets, but the functional significance of this association is poorly understood. Adenovirus mediated transfer of Cav1-GFP in NIH3T3 cells demonstrated that caveolin-1-coated lipid droplets can grow larger than caveolin-1 devoid lipid bodies suggesting the importance of caveolins in determining the size of lipid droplets. This study provided the first detailed characterization of the impact of caveolins on molecular composition and the size of lipid droplets [113].

#### ***Proximity ligation assay***

**Figure 5:**
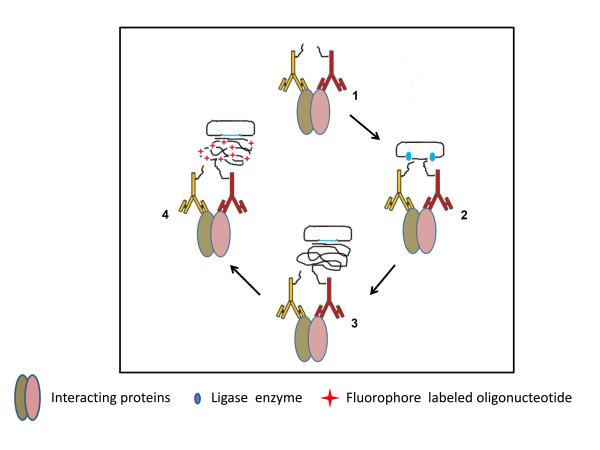
**In situ proximity ligation assay.** (1) Recognition and binding of oligonucleotide labeled antibodies to interacting target proteins. (2) Formation of covalently joined circular oligonucleotide by ligase reaction: If the two probes are in close proximity, addition of two linear oligonucleotide leads to the formation of a covalently joined circular oligonucleotide molecule with the help of a ligase enzyme. (3) Amplification via Rolling circle mechanism: One of the probes linked to antibody act as a primer and addition of DNA polymerase yields long single stranded concatemeric DNA molecule composed of complements of the circular DNA strands formed in the ligase reaction. (4) Fluorophore labeling for detection: The amplified product is easily detected by addition of complementary oligonucleotides labeled with fluorophore.

The most frequently employed tools (ELISA, Western Blot, FRET, FRAP co-immunoprecipitation) to study protein expression or protein-protein interactions rely on the use of antibodies tagged with either fluorophores or enzymes. The sensitivity of these techniques differs significantly, and the detection of proteins at low expression levels still remains challenging. The proximity ligation assay is a relatively inexpensive quantitative technique with high sensitivity. The technique is developed on the principles of antibody targeted assay, split reporter assay and polymerase based amplification of oligonucleotides. In this assay, the target proteins are recognized by specific antibodies (two or more) conjugated with short oligonucleotide strand. In case these probes are in near proximity, the oligonucleotides can be ligated by addition of complementary oligonucleotide and ligase enzyme and subsequently amplified using PCR or rolling circle amplification. This leads to the formation of more than 1000 copies of the complement of 100 bp in one hour using the phi29 polymerase [114]. The incorporation of multiple fluorescently labeled oligonucleotides in the polymerase catalyzed reaction causes amplification of the signal resulting in high sensitivity. Apart from protein expression and protein-protein interaction, this highly versatile technique can also be adapted to detect post-translational modifications, co-localization and interaction of proteins with other biomolecules (Figure 5). PLA was recently used to demonstrate that the MYC inhibitor 10058-F4 could prevent MYCN/Max interaction in situ and caused accumulation of lipid droplets in tumor cells [115].

## Concluding remarks

The availability of a wide range of fluorescent proteins and organelle specific stains provides unprecedented opportunities for microscopic studies of diverse cellular processes including protein-protein interactions. However, for the visualization of certain cell components in live cells such as lipid droplets options are currently limited to organic dyes, i.e. BODIPY, Nile red and Oil red O. The emission spectrum of these dyes overlaps with that of GFP and RFP; thus, it is not feasible to use them in combination for multi-color imaging. There is unmet need for the development of a new lipophilic dye which can be spectrally resolved from commonly used fluorophores. Monodansylpentane, a cadaverin family dye with blue emission spectra, is the appropriate substitute for the red dyes for lipid staining. Furthermore, this can be used along with far wavelength fluorescent proteins to obtain better insight into the lipid metabolism. Discovery of novel proteins involved in the lipid droplet metabolism and their fusion with fluorescent proteins that can be spectrally resolved will help researchers to delineate the process of biogenesis of lipid droplets under live cell conditions. The advancements in culture systems to ensure long term metabolic activity of hepatocytes, the development of suitable transfection methods and reporter assays will be instrumental for the success of live cell imaging experiments.

## Competing interests

The authors declare that they have no competing interests.

## Authors’ contributions

JB initiated the review. All authors contributed to the writing and approved the final manuscript.
